# Detection of tissue factor–positive extracellular vesicles using the ExoView R100 system

**DOI:** 10.1016/j.rpth.2023.100177

**Published:** 2023-05-16

**Authors:** Joshua M.J. Price, Yohei Hisada, Jon Hazeldine, Victoria Bae-Jump, Thomas Luther, Nigel Mackman, Paul Harrison

**Affiliations:** 1 Institute of Inflammation and Ageing, University of Birmingham, Birmingham, United Kingdom; 2 Division of Hematology and Oncology, UNC Blood Research Center, Department of Medicine, University of North Carolina at Chapel Hill, Chapel Hill, North Carolina, USA; 3 Division of Gynecologic Oncology, University of North Carolina at Chapel Hill, Chapel Hill, North Carolina, USA; 4 Department of Medicine, Lineberger Comprehensive Cancer Center, University of North Carolina at Chapel Hill, Chapel Hill, North Carolina, USA; 5 Institute of Pathology, Technical University Dresden, Dresden, Germany

**Keywords:** antigens, CD142, extracellular vesicles, ovarian cancer, tissue factor, trauma

## Abstract

**Background:**

Tissue factor (TF) is essential for hemostasis. TF-expressing extracellular vesicles (TF^+^ EVs) are released in pathological conditions, such as trauma and cancer, and are linked to thrombosis. Detection of TF^+^ EV antigenically in plasma is challenging due to their low concentration but may be of clinical utility.

**Objectives:**

We hypthesised that ExoView can allow for direct measurement of TF^+^ EV in plasma, antigenically.

**Methods:**

We utilized the anti-TF monoclonal antibody 5G9 to capture TF EV onto specialized ExoView chips. This was combined with fluorescent TF^+^ EV detection using anti-TF monoclonal antibody IIID8-AF647. We measured tumor cell-derived (BxPC-3) TF^+^ EV and TF^+^ EVs from plasma derived from whole blood with or without lipopolysaccharide (LPS) stimulation. We used this system to analyze TF^+^ EVs in 2 relevant clinical cohorts: trauma and ovarian cancer. We compared ExoView results with an EV TF activity assay.

**Results:**

BxPC-3-derived TF^+^ EVs were identified with ExoView using 5G9 capture with IIID8-AF647 detection. 5G9 capture with IIID8-AF647 detection was significantly higher in LPS+ samples than in LPS samples and correlated with EV TF activity (*R*^2^ = 0.28). Trauma patient samples had higher levels of EV TF activity than healthy controls, but activity did not correlate with TF measurements made by ExoView (*R*^2^ = 0.15). Samples from patients with ovarian cancer have higher levels of EV TF activity than those from healthy controls, but activity did not correlate with TF measurement by ExoView (*R*^2^ = 0.0063).

**Conclusion:**

TF^+^ EV measurement is possible in plasma, but the threshold and potential clinical applicability of ExoView R100, in this context, remain to be established.

## Essentials

• Tissue factor (TF) can be expressed on extracellular vesicles (EVs).
• TF-expressing EVs (TF^+^ EVs) are challenging to measure directly but may hold clinical utility.
• ExoView can successfully measure TF^+^ EV in plasma and cell culture.
• ExoView TF^+^ EV measurements correlate with EV TF activity for *in vitro* stimulated samples.

## 1 Introduction

Tissue factor (TF/CD142) is a transmembrane protein essential for normal hemostasis [1,2]. TF binds factor (F)VII and FVIIa, and the TF-FVIIa complex activates FIX and FX, thereby promoting the formation of the prothrombinase complex, which in turn cleaves prothrombin to form thrombin. TF is primarily expressed around blood vessels and at body surfaces [3]. Under pathological conditions, levels of TF increase in the circulation. For instance, TF can be found on circulating monocytes [4], on extracellular vesicles (EVs) [5], and as a soluble, alternatively spliced form that has low procoagulant activity [6]. TF^+^ EVs are released into the circulation in cancer following trauma, especially traumatic brain injury, where they can activate coagulation [[7], [8], [9]]. TF^+^ EVs have been linked with thrombosis in COVID-19 [[10], [11], [12], [13], [14]].

Direct antigenic measurement of TF in plasma has been reported. However, the low abundance of TF (<20 fM [1], <2 pM [15]) in healthy individuals typically falls below the detection limit of these assays [[15], [16], [17], [18]]. Furthermore, the background signal of these assays is sometimes high in plasma [15,19,20]. These issues are further compounded by the specificity of the antibodies used and the type of TF standard employed [21]. TF activity assays are highly sensitive [22] but typically require the isolation of EVs from the plasma [23]. Furthermore, TF activity assays cannot measure encrypted TF, which may form a pool of physiologically latent TF activity. Despite these problems, the direct measurement of TF in plasma may hold clinical utility to identify individuals at the risk of thrombosis [5,24,25].

Accurate and specific measurement of TF^+^ EVs is challenging [26,27]. In addition to TF measurement, EV measurement has specific limitations [28]. EVs are small and often released into complex biofluids. Their small size and physiological abundance necessitate specialist knowledge and measurement devices for accurate purification and enumeration. Even then, the vast majority of EVs fall under the detection level of conventional flow cytometry [28]. Nanoparticle tracking analysis (NTA) allows for measurement of EV size and concentration. However, NTA is highly user-dependent and samples typically require purification of EVs [29]. NTA also has limited capacity to measure specific subpopulations of EV based on marker expression, thereby reducing utility in TF^+^ EV measurement.

Nieuwland et al. [26] and Gardiner et al. [27] highlighted the specific challenges of TF^+^ EV research and suggested a roadmap toward standardizing TF^+^ EV measurements. Measuring TF-expressing EVs necessitates a highly sensitive measurement methodology, probably in combination with a purification process. In the absence of a gold standard, comparison to activity assays is required [26,27]. Preanalytical variables are paramount; it is important to use well-accepted positive and negative controls to establish the accuracy of an assay [26,27]. An assay that can differentiate positive and negative controls while correlating with other measurement methods (eg, activity) is a candidate assay for application to clinical samples [26,27]. Increasingly, antibody-based capture systems have been shown to have great utility in capturing EVs based on their marker expression [[30], [31], [32]]. Capture-based systems act as the purification platform as well as the measurement platform, allowing for direct capture of EVs with minimal sample manipulation in complex biofluids, such as plasma [31]. Capture-based systems potentially facilitate detection of acute but biologically relevant EV populations [33].

The ExoView R100 system (Unchained Labs) is a chip-based system that captures EV based on their surface marker expression on printed antibody capture spots [34]. ExoView incorporates the single-particle interferometric reflectance imaging sensor technology for high-resolution sizing and quantification of EVs [34,35]. The capture spots are printed in triplicate, and the recent development of ExoFlex chips allows customized capture through addition of user-chosen antibodies. ExoView also incorporates fluorescent labeling of captured EVs. [31,32,[36], [37], [38]].

Here, we investigated the potential of ExoView technology for direct capture and subsequent fluorescent labeling of TF-expressing EVs derived from the human pancreatic cell line BxPC-3, in platelet-free plasma (PFP) containing either TF^−^ EV or TF^+^ EV, in PFP of trauma patients and platelet-poor platelet (PPP) of ovarian cancer patients. We also show how these measures compare to a well-established TF activity assay [22].

## 2 Methods

### 2.1 Volunteers

Healthy volunteers were recruited from the *University of Birmingham Research Laboratories*, Queen Elizabeth Hospital. Blood samples were collected from trauma patients during prehospital emergency evacuation, prior to receipt of prehospital blood products (research ethics committee approval: Brain Biomarkers After Trauma Cohort Study; reference 13/WA/0399). Blood samples were collected from patients with ovarian cancer using a protocol approved by the University of North Carolina at Chapel Hill Institutional Review Board (11-1201). Some of the ovarian cancer samples were used in a previous study [39]. Some of the trauma samples were used in previous studies [40]. Details on clinical cohorts can be found in Supplementary Methods.

Informed written consent was received from all participants. Blood was collected from each participant via antecubital venepuncture using a sterile 21-gauge needle and aseptic technique. Blood was drawn into 3.2% trisodium citrate anticoagulant (9:1 vol/vol) vacutainers (BD) for experiments. Inclusion criteria for healthy controls (HCs) were an absence of medication, an absence of a diagnosed illness, and an absence of known acute episode of infection. HCs were excluded if they were taking any medication, such as steroids, COX-1 inhibitors, and antiplatelet drugs. Trauma participants were recruited as previously described**.** [40] Briefly, on a 24 × 7 basis, prehospital emergency care teams acquired blood samples from adult trauma patients (aged ≥18 years) with a suspected injury severity score of ≥8 within 1 hour of injury (defined as the time of phone call to emergency services). Owing to their injury severity, patients were often unable to provide informed consent to enroll. Recruitment occurred under the guidance of the Mental Health Capacity Act for research in emergency situations, in accordance with the Declaration of Helsinki. Where patients lacked capacity, a written agreement for study participation was sought from a legal consultee, with written consent obtained from the patient after they regained capacity. In cases in which the patient did not regain capacity to consent, data were retained in accordance with the legal consultee’s assent. Where consent was withdrawn, samples and data were destroyed.

We prospectively recruited patients with epithelial ovarian cancer between May 2017 and June 2019 at the University of North Carolina. Blood samples were collected from patients who provided written consent using a protocol approved by the Institutional Review Board of the University of North Carolina at Chapel Hill (11-1201).

### 2.2 Preparation of PFP

PFP negative controls were generated from HCs by centrifugation of trisodium citrate anticoagulated whole blood at 2000 × *g* for 20 minutes, followed by 13,000 × *g* for 2 minutes. Negative control plasma was also prepared immediately after blood collection [2,22,26]. For TF^+^ control sample generation, whole blood was stimulated with lipopolysaccharide (LPS) (10 mg/mL) (L2887, Sigma-Aldrich) for 5 hours at 37 °C, followed by double centrifugation. Whole blood samples with and without stimulation were assessed by flow cytometry. Following stimulation, positive control PFP was generated. PFP samples from trauma patients were prepared by double centrifugation (2000 × *g* for 20 minutes, 13,000 × *g* for 2 minutes) of trisodium citrate anticoagulated whole blood. PPP was prepared from trisodium citrate anticoagulated whole blood of patients with ovarian cancer by centrifugation at 1500 × *g* for 15 minutes. PFP and PPP were aliquoted and stored at −80 °C.

### 2.3 BxPC-3 cell culture

BxPC-3 cells (American Type Culture Collection [ATCC]) were thawed upon receipt for 10 minutes at 37 °C and resuspended in complete growth medium (CGM) (RPMI 1640 supplemented with glutamine and penicillin/streptomycin, 10% fetal calf serum [FCS]) for a final total volume of 12 mL split across 2 T25 flasks. CGM (10% FCS) was replaced every 3 days. Upon achieving confluence, cells were removed with trypsin and placed into T150 flasks.

For experiments, when T150 flasks reached 75% confluence, CGM was replaced with serum-free CGM. After 3 days, cell culture supernatant was collected. Conditioned media was spun at 300 × *g* for 5 minutes to remove cellular debris and subsequently double centrifuged at 2000 × *g* for 20 minutes and 13,000 × *g* for 2 minutes. Cell culture supernatant samples were then stored at −80 °C.

### 2.4 TF antibody choice

Human TF is a 263-amino–acid protein that comprises an extracellular domain (amino acids 1-219), a transmembrane domain (amino acids 220-242), and a cytoplasmic domain (amino acids 243-363) [41]. Several groups have generated monoclonal antibodies against human TF [[42], [43], [44], [45], [46], [47]]. These antibodies can be broadly divided into 3 groups: group 1, antibodies that bind to the N-terminal region of the extracellular domain of TF and compete with FVII/FVIIa; group 2, antibodies that bind to the C-terminal region of the extracellular domain of TF that can bind to either free TF or TF complexed with FVII/FVIIa and interfere with substrate binding; group 3, antibodies that bind to the C-terminal region of the extracellular domain of TF close to the transmembrane membrane. In general, antibodies in group 2 are more potent inhibitors of the TF/FVIIa complex than antibodies in group 1, and antibodies in group 3 do not inhibit TF procoagulant activity [[47], [48], [49], [50], [51]]. We used HTF-1 to inhibit TF activity. This antibody has been used to inhibit TF activity in numerous studies and is commercially available [22,42]. HTF-1 binds to a region of the N-terminal domain of the extracellular domain of TF that binds FVII/FVIIa [47,48]. For the capture and detection of TF, we selected 1 antibody from group 2 and 1 antibody from group 1. The anti-TF antibody we selected from group 2 for the capture of TF was 5G9. 5G9 is commercially available (PABW-121, Creative Biolabs). This is a well-characterized antibody that binds to the C-terminal domain of the extracellular domain of TF (amino acids 181-214, specifically amino acids 156, 169, 200, and 201) [48,51]. The anti-TF antibody we selected from group 1 for detection of TF was IIID8. The choice of IIID8 was based in part on data that we obtained by flow cytometry. IIID8 is commercially available (4509, American Diagnostica) and binds to the N-terminal domain of the extracellular domain of TF (amino acids, 1-25) [48,49]. IIID8 has been used previously for immunofluorescence, flow cytometry, and western blot [[52], [53], [54], [55], [56]].

### 2.5 Monocyte flow cytometry

Two hundred microliters of unstimulated whole blood or LPS-stimulated blood (5 hours, 10 μg/mL) was stained with 5 μL of CD14-FITC (clone TUK4; Dako) and 1 μL of anti-TF antibody (IIID8-AF6F7, 5G9-AF647, or VIC12-AF647) (1:200) [49] for 20 minutes at room temperature. In some of these experiments, there was a delay in processing the unstimulated samples. Stained whole blood was incubated for 20 minutes at room temperature. Following incubation, 200 μL of whole blood was lysed with 2 mL of 1× lysis buffer (BD FACS lysis buffer) and incubated for 10 minutes in the dark. Following lysis, cells were centrifuged at 250 × *g* for 5 minutes and resuspended in 200 μL of phosphate-buffered saline. Monocytes were gated on by their classical forward scatter and side scatter. Monocyte events (N = 2500) were then identified using CD14-FITC and TF-AF647 median fluorescence intensity (MFI) and percentage of TF^+^ events were measured.

### 2.6 NTA

All samples were diluted in phosphate-buffered saline to a final volume of 1 mL. Optimal measurement concentrations were determined by pretesting the optimal particle per frame value (20-100 particles per frame). The following settings were set according to the manufacturer’s software manual (NanoSight NS300 User Manual, AN0541-01-EN-00, 2017): camera level was increased until all particles were distinctly visible not exceeding a particle signal saturation over 20% (cell line-derived EVs: level 16). The optimal detection threshold was determined to include as many particles as possible with the restrictions that 10 to 100 red crosses were counted, whereas only <10% were not associated with distinct particles. Blue cross count was limited to 5. Autofocus was adjusted so that indistinct particles were avoided. For each measurement, five 1-minute videos were captured under the following conditions: cell temperature, 25 °C; syringe speed, 40 μL/s. After capture, the videos were analyzed using NanoSight software NTA 3.1 Build 3.1.46 with a detection threshold of 8. Hardware: embedded laser: Blue405; camera: sCMOS. The number of completed tracks in NTA measurements was always greater than the proposed minimum of 1000 to minimize data skewing based on single large particles.

### 2.7 Measurement of EV TF activity

Measurement of TF activity was conducted as previously described [22].

### 2.8 Calibrated automated thrombography

Thrombin generation was assessed using calibrated automated thrombography (CAT) as described by Hemker et al. [57] Briefly, 96-well, round-bottomed plates were prepared, with 4 wells containing 80 μL of sample and 20 μL of either microparticle (MP) reagent or thrombin calibrator (Stago) added in duplicate wells. Plates were then incubated for 10 minutes at 37 °C, after which 20 μL of fluorogenic substrate for thrombin (Z-Gly-Gly-Arg-aminomethylcoumarine) plus calcium chloride (FLUCA) reagent (Stago) was automatically added to initiate thrombin generation. The thrombin calibrator (Stago) contains a known concentration of thrombin, which was used to calibrate each sample to internally control for quenching and nonlinearity in individual samples [58]. After sample activation, the generation of fluorescence (excitation and emission wavelengths of 360 nm and 460 nm, respectively) was continuously monitored for up to 1 hour in all wells using a fluorescent plate reader (Fluoroskan Ascent; Thermo Scientific). Thrombin generation parameters were then automatically calculated by the Hemker software (Thrombinoscope software, version V5.0.0.742; Stago). In some experiments, LPS^+^ PFP was double centrifuged at 20,000 × *g* to pellet and remove EV. Additional conditions included either HTF-1 (final concentration, 9.15 μg/mL) or control mouse IgG pretreatment of LPS^+^ PFP prior to the 10-minute incubation period. The 20,000 × *g* double centrifugation was based on the TF activity assay protocol [22]. The purpose was to measure thrombin generation in the absence of EV generated by LPS stimulation in PF.

### 2.9 ExoView

EVs were captured and measured by the ExoView R100 reader (Unchained Labs). ExoFlex chips (Unchained Labs) were used for all samples. Chips were arrayed with capture antibody (in triplicate) against mouse IgG, anti-CD81, peptide material labeled “ExoFlex 1” (allowing for 5G9 capture). ExoFlex chips allow for the addition custom antibodies via antibody-linker conjugation to peptide-linker “ExoFlex 1.” ExoFlex 1 linker binds to ExoFlex 1 on the surface of ExoView (Unchained Labs) chips. The details of antibody processing and conjugation to ExoFlex Linker 1 are described in Supplementary Methods under the heading “TF antibody processing and conjugation.” To allow binding of ExoFlex 1 linker-conjugated antibody (5G9) to “ExoFlex 1,” linker-conjugated antibodies were allowed to equilibrate to room temperature and were then diluted 1:100 in solution A (Unchained Labs). For some experiments, a second peptide material was used labeled “ExoFlex 2.” This allowed for conjugation of anti-TF antibody (HTF-1). Chips were placed in separate wells of a 24-well plate. Thirty-five μL of diluted antibody-linker conjugate was incubated on the surface of each chip for 30 minutes at room temperature. To remove any unbound material and prepare chips for sample incubation, they were washed following manufacturer recommendation with kit-provided solutions. Briefly, 1 mL of solution A (Unchained Labs) was added to each well, and 750 μL of solution within the wells was then discarded. Thereafter, 750 μL of solution B (Unchained Labs) was added to each well, and 750 μL of solution within the wells was then discarded and replaced with 750 μL of 1-μm filtered distilled water. Chips were carefully removed and placed in Petri dishes (10-cm diameter) containing 1-μm filtered distilled water. Chips were washed and dried and then placed in separate wells of a new 24-well plate.

PFP was diluted 1:25 in an incubation solution and cell culture supernatant was diluted 1:2 in the incubation solution. Thereafter, 50 μL of diluted sample was applied to each chip. Distilled water was added to the void spaces between wells (to provide humidity), the plate was sealed and incubated for 16 hours at room temperature in the dark. Chips were then washed 3 times with solution A. After each wash, the plate was shaken at 500 rpm (LSE Digital Microplate Shaker; Corning) for 3 minutes. Following the final wash, 250 μL of kit-provided blocking solution (Unchained Labs) and IIID8 (anti-TF)-AF647(final dilution, 1:500) were added to each well. The plates were incubated for 1 hour at room temperature in the dark. Wells containing chips were then washed 5 times, the first wash in solution A (Unchained Labs), the next 3 washes in solution B (Unchained Labs), and a final wash in 0.1-μm filtered distilled water. Chips were carefully removed and placed in petri dishes (10 cm diameter) containing 1-μm filtered distilled water. Chips were washed, dried, and imaged using the ExoView R100 reader using ExoViewer 3.14 software. The data were exported using ExoView Analyser 3.0 with fluorescence gating based on control mouse IgG capture. Sizing thresholds were set from a diameter of 50 to 200 nm.

### 2.10 Statistical Analysis

Statistical analysis and figures were generated using RStudio. For all data sets, normality was tested. For all data sets where >2 groups were compared, Kruskall-Wallis tests were performed, followed by Wilcoxon signed-rank tests where sample groups were related and Wilcoxon rank-sum tests where sample groups were unrelated. The Holm-Bonferroni method was applied to correct for multiple comparisons where appropriate. Wherever data sets contained 2 samples and were normally distributed, *t*-tests were applied. Where data were not normally distributed, Wilcoxon signed-rank test was used to determine significance. The predefined level of significance was set at 5% (α level = 0.05). In the results section, data are reported as mean, unless otherwise indicated.

## 3 Results

### 3.1 TF expression by LPS-stimulated monocytes

**Figure 1:**
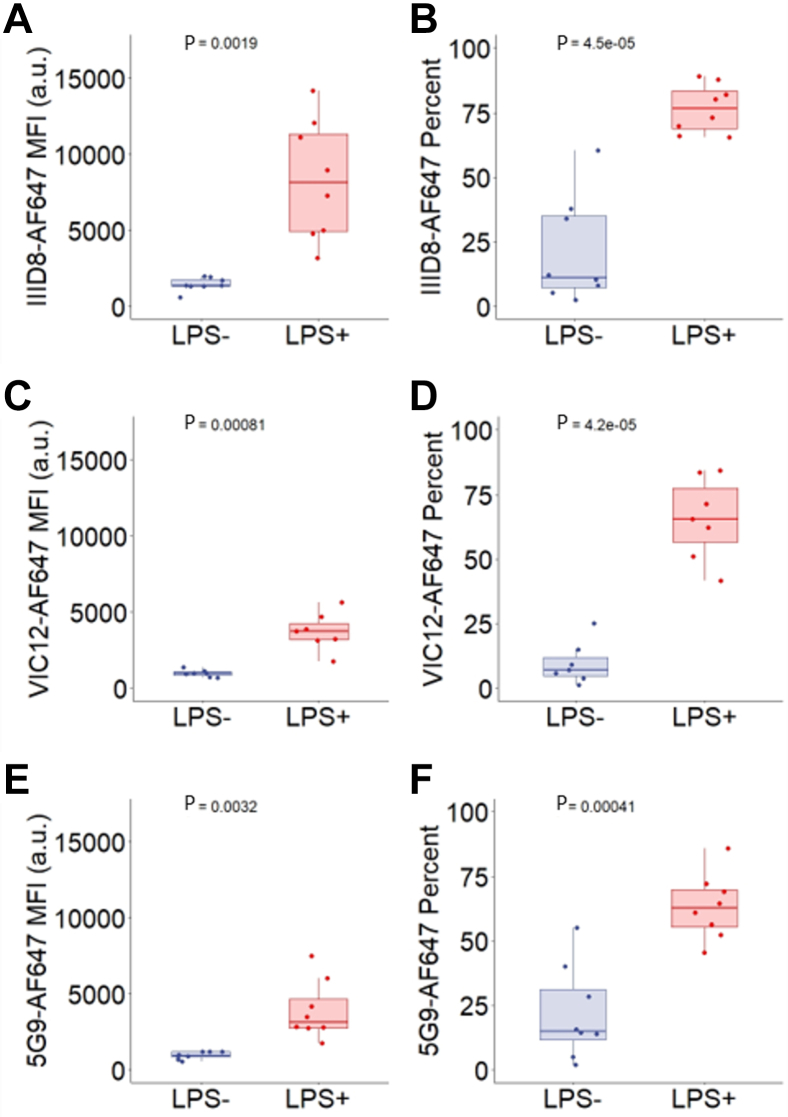
Tissue factor (TF) expression by lipopolysaccharide (LPS)-stimulated CD14+ monocytes. (A) Anti-TF antibody (IIID8-AF647) median fluorescence intensity, *P* = .0019, N = 8. (B) Percentage of anti-TF antibody (IIID8-AF647) monocytes expressing TF, *P* = .000045, N = 8. (C) Anti-TF antibody (VIC12-AF647) median fluorescence intensity, *P* = .00081, N = 7. (D) Percentage of anti-TF antibody (VIC12-AF647) monocytes expressing TF, *P* = .000045, N = 7. (E) Anti-TF antibody (5G9-AF647) median fluorescence intensity, *P* = .0032, N = 8. (F) Percentage of anti-TF antibody (5G9-AF647) monocytes expressing TF, *P* = .00041, N = 8. LPS stimulation occurred by 10-μg/mL LPS stimulation under gentle agitation for 5 hours at 37 °C. CD14- FITC^+^ positive monocyte events were selected. MFI, median fluorescence intensity.

The gating strategy used to identify TF^+^ monocytes is shown in Supplementary Figure S1. After gating based on forward and side scatter, doublets were removed and CD14^−^ FITC^+^ positive events were selected. Thereafter, 2500 CD14^+^ monocytes were acquired. Whole blood was stained with TF(IIID8)-AF647, TF(VIC12)-AF647, or TF(5G9)-AF647. TF antibodies were conjugated to AF647 in-house. In order to validate if conjugation was successful, LPS stimulation of whole blood was conducted. This is a well-established method to generate TF^+^ monocytes [19]. Figure 1A, C, and E shows that the MFI for TF-AF647 increases following LPS stimulation with all TF antibodies tested (1449 a.u. to 8239 a.u. with TF(IIID8) [*P* < .01], 969 a.u. to 3725 a.u. with TF(VIC12) [*P* < .001], 950 a.u. to 3921 a.u. with TF(5G9) [*P* < .01]). Figure 1B, D, and F shows that LPS stimulation of whole blood increased the percentage of TF^*+*^ monocytes with all 3 anti-TF antibodies (from 21.3% to 76.7% with TF(IIID8) (*P* < .001), 9.5% to 65.6% with TF(VIC12) (*P* < .001), and from 21.8% to 63.3% with TF(5G9) (*P* < .001). These data indicate that all conjugations were successful. TF(IIID8)-AF647 showed the greatest discrimination when assessed by MFI and was, therefore, chosen as the antibody for fluorescent EV detection by ExoView.

### 3.2 Characterization of BxPC-3-derived EVs by NTA and ExoView TF detection

**Figure 2:**
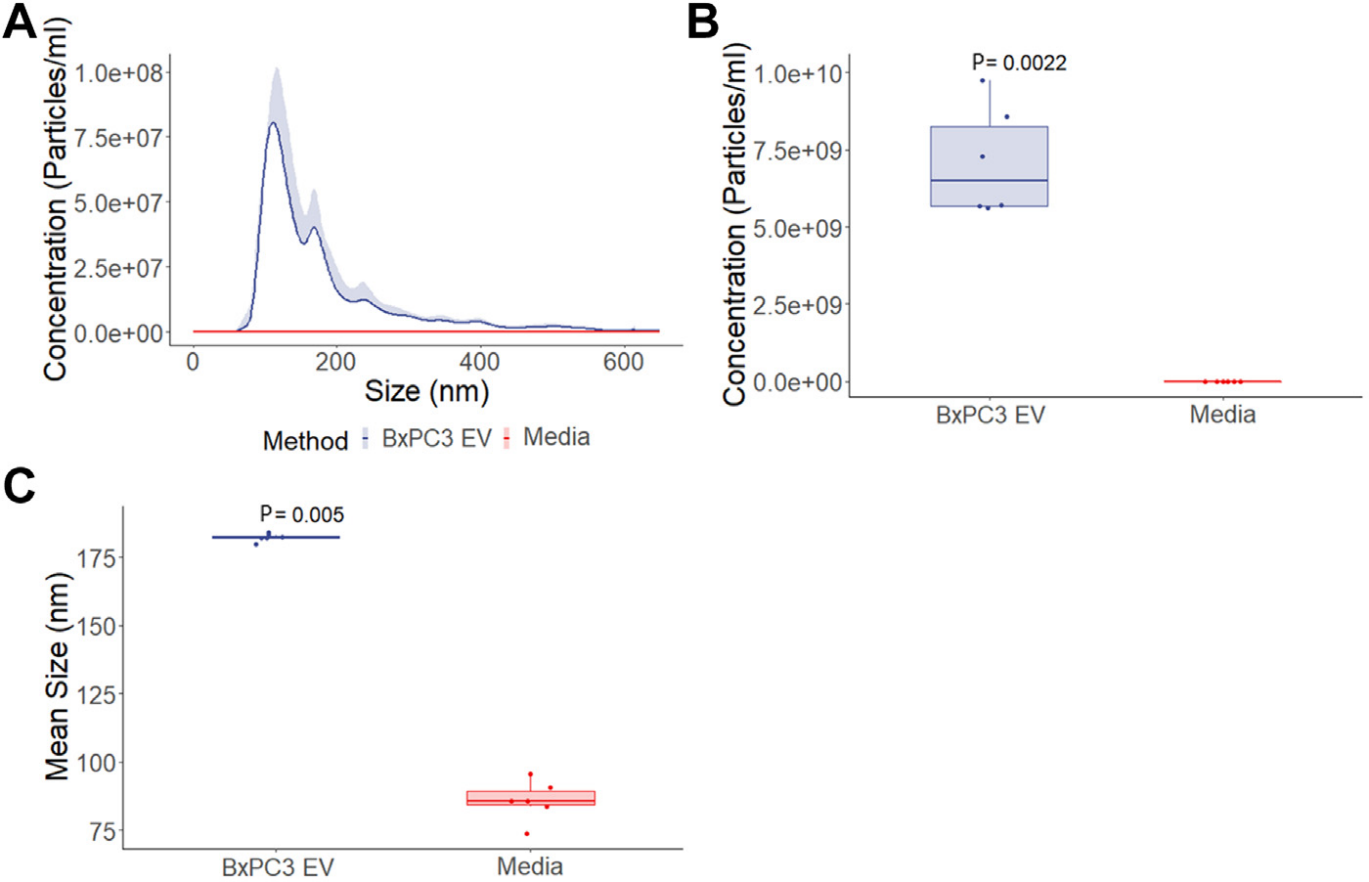
Characterization of BxPC-3–derived extracellular vesicles using nanoparticle tracking analysis (NTA). (A) NTA size and concentration distribution of particles in culture supernatant from serum-starved BxPC-3 cells compared to media, N = 6, each with 5 replicates. (B) NTA particle concentration in culture supernatant from serum-starved BxPC-3 cells compared to media, *P* = .0022, N = 6. (C) NTA mean size comparison of particles in culture supernatant from serum-starved BxPC-3 cells compared to media, *P* = .005, N = 6. EV, extracellular vesicle.

Figure 2 shows NTA characterization of EV derived from the culture supernatant of serum-starved BxPC-3 cells compared with serum-free media control. Figure 2A shows the size and concentration distribution of EVs. Figure 2B shows BxPC-3-derived EV from culture supernatant (7.1 × 10^9^ particles/mL) compared with serum-free media control (3.1 × 10^5^ particles/mL) (*P* < .005). Figure 2C shows that BxPC-3 derived EVs isolated from culture supernatant are larger than those of the serum-free media control (*P* < .005). NTA is not capable of directly identifying TF^+^ EVs; however, these data do show that there are EVs present in BxPC-3 cell culture supernatant.

**Figure 3:**
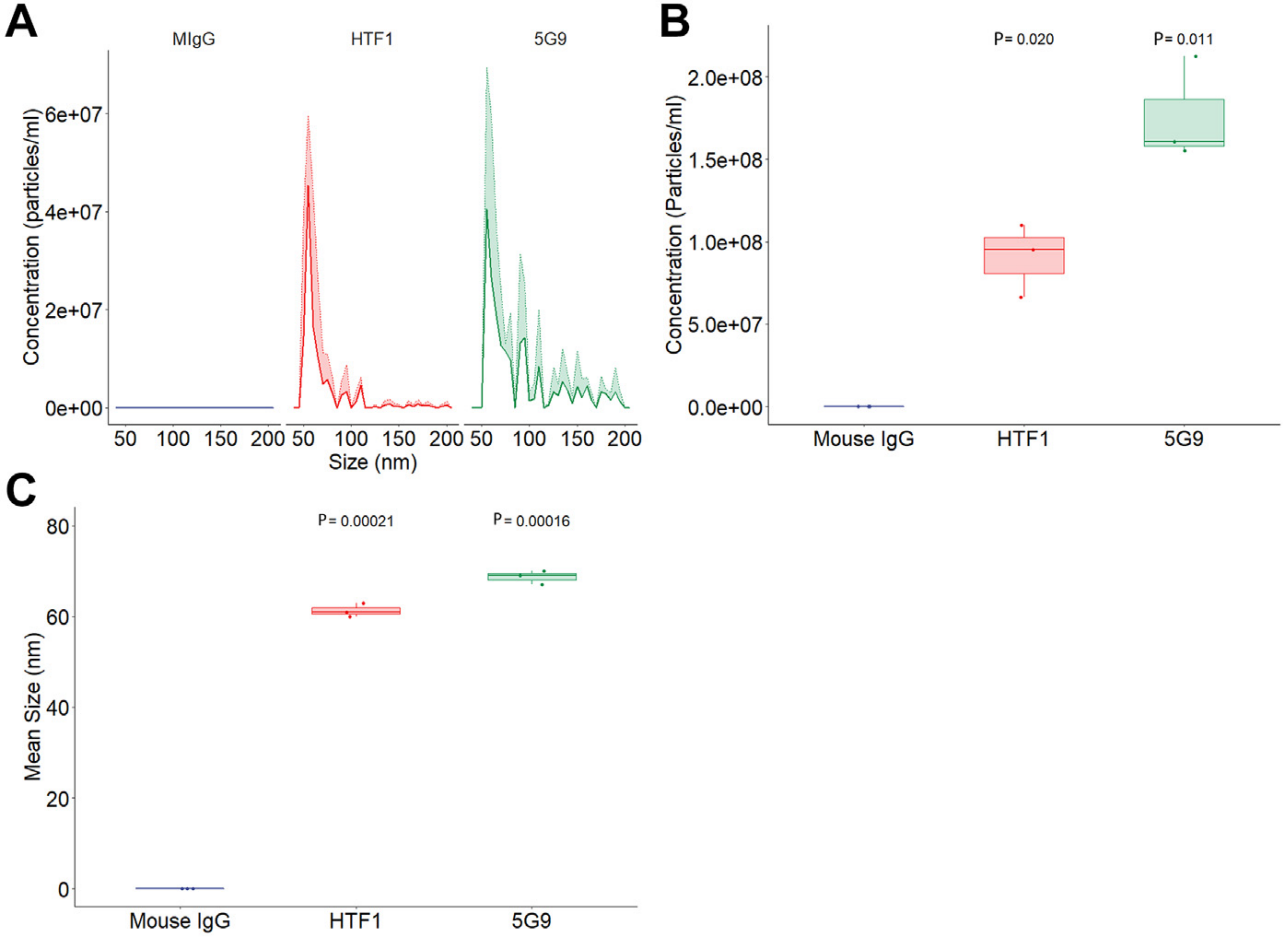
Tissue factor (TF)^+^ BxPC-3–derived extracellular vesicles captured using ExoView technology. (A) ExoView interferometric imaging size and concentration distribution of particles in culture supernatant from serum-starved BxPC-3 cells stratified by capture marker: mouse IgG control, anti-TF antibodies (HTF1 and 5G9), N = 3, each with 3 replicates. (B) ExoView interferometric imaging concentration of particles in culture supernatant from serum-starved BxPC-3 cells stratified by capture marker compared to mouse IgG capture, HTF1 capture *P* = .02, N = 3; 5G9 capture, *P* = .011, N = 3. (C) ExoView interferometric imaging size of particles in culture supernatant from serum-starved BxPC-3 cells stratified by capture marker compared to mouse IgG capture, HTF1 capture, *P* = .00021, N = 3; 5G9 capture, *P* = .00016, N = 3.

ExoView is capable of identifying specific populations of EVs based on 2 overlapping systems: antigenic capture with interferometric imaging and fluorescent antigenic detection on the captured EV. Figure 3 shows ExoView interferometric imaging characterization of EV from the culture supernatant of serum-starved BxPC-3 stratified based on capture antibody: mouse IgG control, anti-TF antibody (HTF1) and anti-TF antibody (5G9). Figure 3A shows the size and concentration distribution of EVs derived from the culture supernatant of serum-starved BxPC-3 cells stratified based on capture antibody. Figure 3B shows that 5G9 captures more EVs than mouse IgG (1.8 × 10^8^ particles/mL vs 0 particles/mL, *P* < .05). Figure 3B also shows that HTF1 captures more EV than mouse IgG (9.1 × 10^7^ particles/mL vs 0 particles/mL, *P* < .05). Figure 3C shows that the size of EVs captured on 5G9 is larger than those captured on an isotype control (68.7 nm vs 0 nm, *P* < .001). The size of EV capture by HTF1 is larger than that on mouse IgG (61.3 nm vs 0 nm, *P* < .001). Having shown that TF^+^ EVs are captured by 5G9, we then utilized the validated TF(IIID8)-AF647 shown in Figure 1 to stain the captured EV.

**Figure 4:**
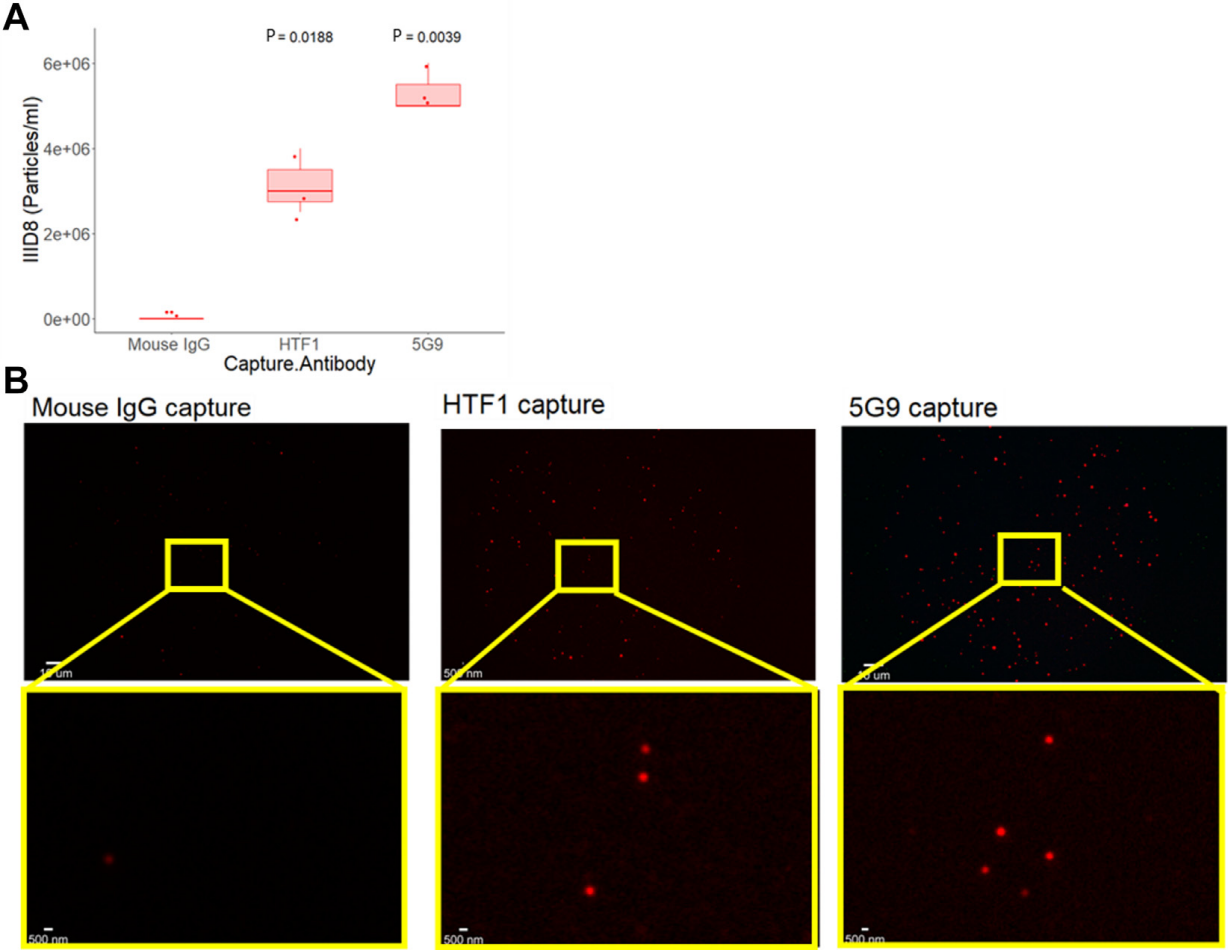
Tissue factor (TF)^+^ BxPC-3–derived extracellular vesicles captured and fluorescently labeled using ExoView technology. (A) ExoView fluorescent labeling (anti-TF antibody: IIID8-AF647) and quantification of particles in culture supernatant from serum-starved BxPC-3 cells stratified by capture marker: mouse IgG control, anti-TF antibody (HTF1) and anti-TF antibody (5G9), N = 3, each with 3 replicates. (B) Representative images of TF (IIID8-AF647)-labeled particles captured on TF capture spots (HTF1, 5G9).

Figure 4 shows the TF(IIID8)-AF647 fluorescent labeling of EVs derived from the culture supernatant of serum-starved BxPC-3 cells captured by isotype control, HTF1 or 5G9. Figure 4A shows that the concentration of TF(IIID8)-AF647–labeled EVs captured on HTF1 is significantly higher than on isotype control capture (3.2 × 10^6^ particles/mL vs 0 particles/mL, *P* < .05). Figure 4A also shows that the concentration of TF(IIID8)-AF647 labeled EVs captured on 5G9 is significantly higher than on isotype control capture (5.3 × 10^6^ particles/mL vs 0 particles/mL, *P* < .005). Figure 4B shows representative images of TF(IIID8)-AF647 fluorescent labeling of BxPC-3 derived EVs stratified by antibody capture spot.

### 3.3 TF activity and ExoView TF detection

**Figure 5:**
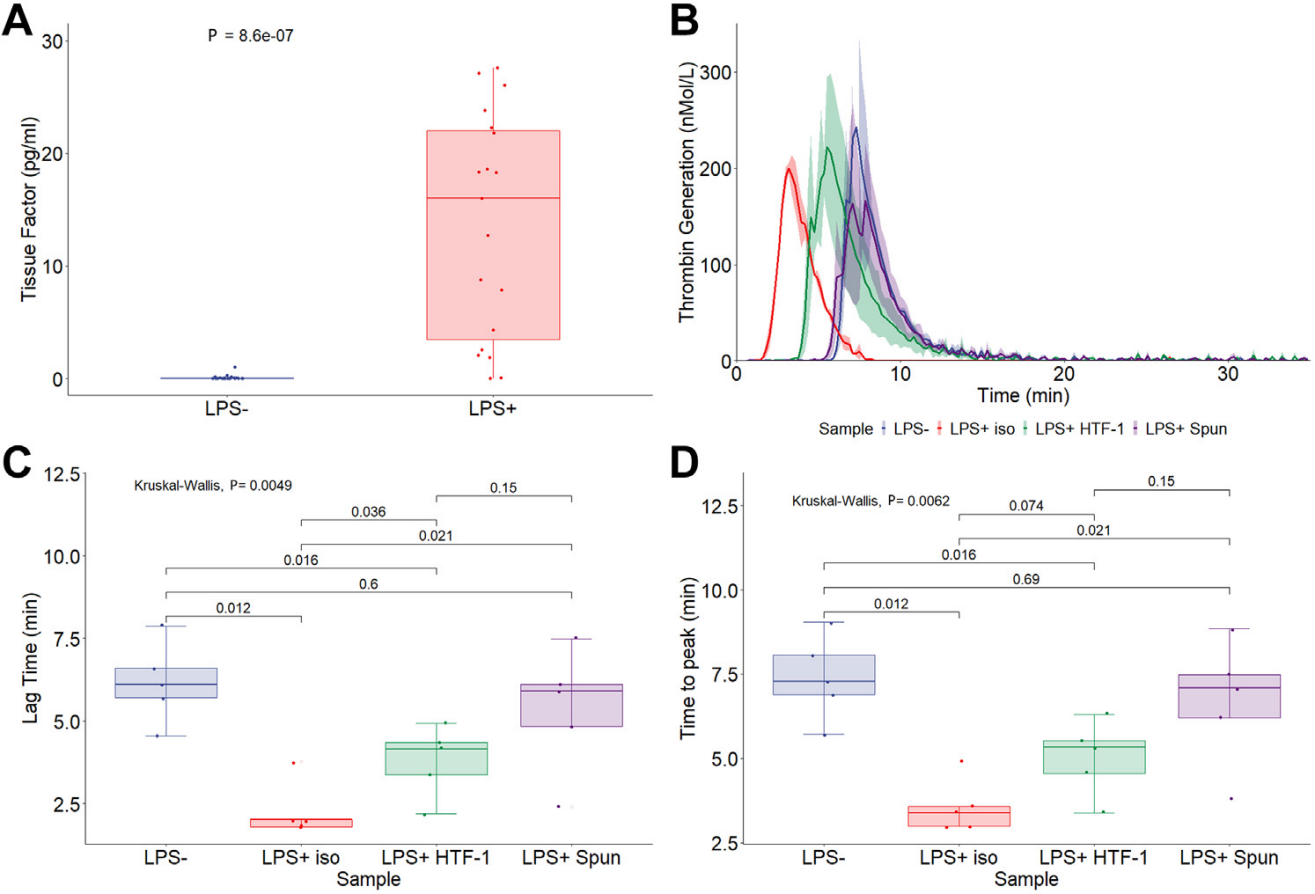
Tissue factor (TF) activity is extracellular vesicle associated. (A) Active TF in platelet-free plasma (PFP) derived from stimulated whole blood (lipopolyscarride [LPS^+^]) compared to unstimulated whole blood (LPS^−^) using an in-house assay, n = 19, *P* = .00000086. (B–D) Calibrated automated thrombography data with microparticle reagent on LPS^−^ PFP, LPS^+^ PFP, and LPS^+^ PFP that underwent double centrifugation at 20,000 × *g* (LPS^+^ spun). (B) Aggregated median line graphs with CI shown. (C) Lag time (minutes), global *P* value = .0049. (D) Time to peak (minutes), global *P* value = .0062.

Cell culture supernatant is a simpler biofluid than plasma and having now established proof of principle that TF detection and capture is possible with ExoView technology, we next sought to assess whether plasma measurement was possible. For plasma TF^+^ EV detection, we first show increased concentrations of active EV TF in PFP derived from LPS-stimulated whole blood compared matched unstimulated whole blood (13.7 pg/mL vs 0.1 pg/mL, *P* < .001). Having shown EV TF activity in EV isolated from LPS^+^ PFP, we next tested the thrombin generation dynamics of LPS^-^ and LPS^+^ plasma measured by CAT. Modifying the EV TF activity assay protocol, we also double spun LPS^+^ plasma at 20,000 × *g* and the plasma supernatant was run on CAT. The inhibitory antibody HTF-1 was used on LPS^+^ plasma to demonstrate that thrombin generation was TF dependent. Figure 5B shows the median and 95% CIs of thrombin generation for LPS^−^, LPS^+^, LPS^+^ spun and LPS^+^ HTF-1 over time measured by CAT. LPS-stimulated whole blood–derived PFP treated with isotype control shows reduced lag time (Figure 5C) compared with matched PFP derived from unstimulated whole blood (2.3 minutes vs 6.2 minutes, *P* < .05). Time to peak (Figure 5D) was also reduced in PFP derived from LPS-stimulated whole blood (3.6 minutes vs 7.4 minutes, *P* < .05).

After double spinning LPS^+^ plasma at 20,000 × *g* (LPS^+^ spun), there was no difference compared to LPS^-^ lag time (5.4 minutes vs 6.2 minutes) and time to peak (6.7 minutes vs 7.4 minutes).

**Figure 6:**
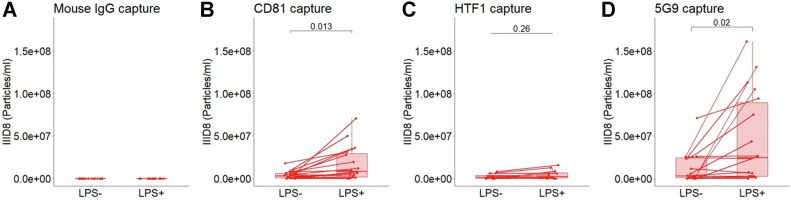
Detection of tissue factor (TF)–positive extracellular vesicles in platelet-free plasma from lipopolysaccharide (LPS)-stimulated blood. (A) ExoView fluorescent extracellular vesicle count (anti-TF antibody (IIID8-AF647)) on platelet-free plasma derived from stimulated whole blood (LPS^+^) compared to unstimulated whole blood (LPS^−^), with results stratified by capture marker: (A) mouse IgG control, (B) anti-CD81 (*P* = .013, N = 21), (C) anti-TF antibody (HTF1) (*P* = .26, n = 12), and (D) anti-TF antibody (5G9) (*P* = .026, N = 18) lines indicate matched LPS^−^ and LPS^+^ samples.

Having established the presence of TF^+^ EV in plasma, we next sought to measure them antigenically, directly from PFP using ExoView. Figure 6 shows TF(IIID8)-AF647 fluorescent labeling of EV from LPS^−^ and LPS^+^ plasma captured by isotype control and 5G9. Figure 6A shows that the concentration of TF(IIID8)-AF647–labeled EV captured on isotype control are not different between LPS^−^ and LPS^+^ samples. Figure 6B shows a higher concentration of CD81 captured, TF(IIID8)-AF647–labeled EV in LPS^−^ than LPS^+^ PFP (4.1 × 10^6^ particles/mL vs 1.6 × 10^7^ particles/mL, *P* < .05). Figure 6C shows no difference in concentration of HTF1 captured, TF(IIID8)-AF647–labeled EV in LPS^−^ compared with that in LPS^+^ PFP (2.3 × 10^6^ particles/mL vs 4.4 × 10^6^ particles/mL). Figure 6D shows a higher concentration of 5G9 captured, TF(IIID8)-AF647–labeled EV in LPS^−^ compared with that in LPS^+^ PFP (4.6 × 10^7^ particles/mL vs 1.2 × 10^7^ particles/mL, *P* < .05). Supplementary Figure S2 shows representative images of LPS^−^ and LPS^+^ fluorescent TF(IIID8)-AF647 stratified by capture antibody.

**Figure 7:**
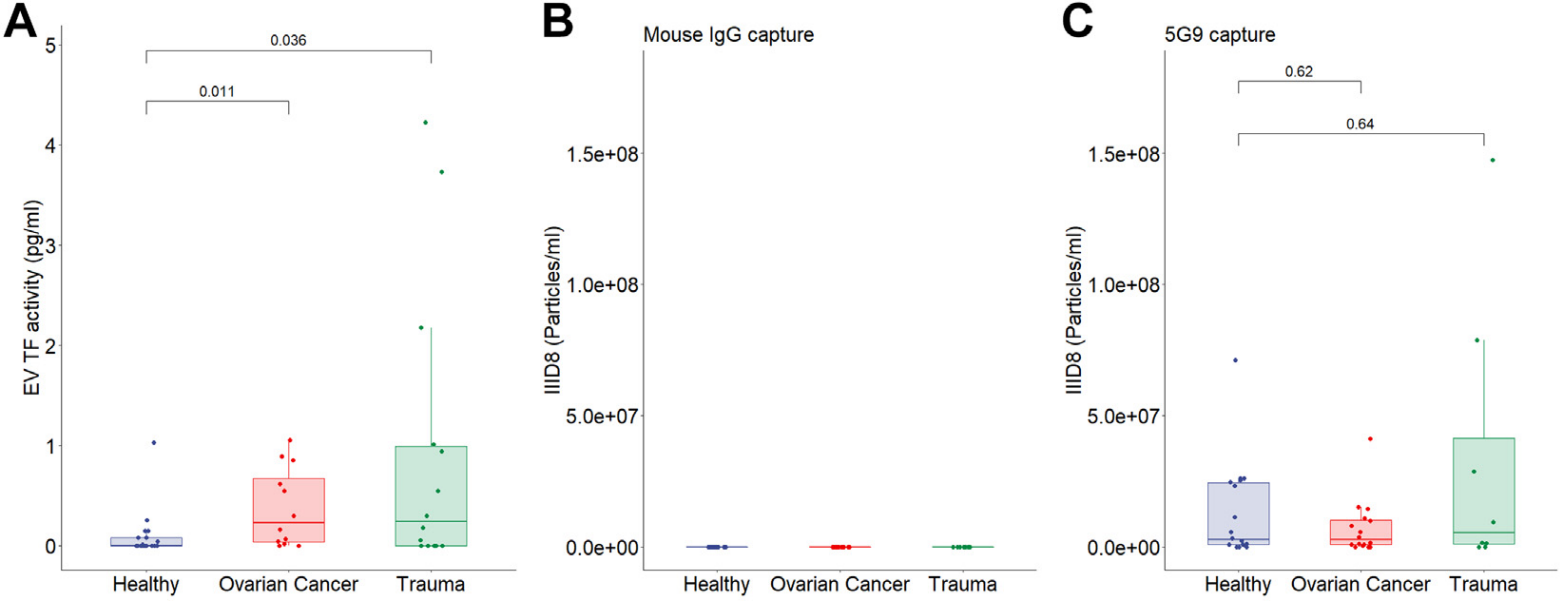
Tissue factor (TF) activity and ExoView measurement in trauma and ovarian cancer. (A) Active extracellular vesicle TF in platelet-free plasma derived from trauma patients within 1 hour of injury (*P* = .0363) and patients with ovarian cancer (*P* = .0037) compared against healthy controls using an in-house assay. (B–C) ExoView fluorescent extracellular vesicle count (anti-TF antibody [IIID8-AF647]) on platelet-free plasma derived from healthy controls (N = 18), trauma patients within 1 hour of injury (*P* = .67, N = 8), and patients with ovarian cancer (*P* = .47, N = 12), with results stratified by capture marker. Results are compared to healthy controls, (B) mouse IgG control, and (C) anti-TF antibody (5G9). EV, extracellular vesicle.

We next wanted to determine whether ExoView antigenic detection could detect TF^+^ EV in clinically samples. For this purpose, a trauma cohort and an ovarian cancer cohort were used. Figure 7A shows increased concentrations of EV TF activity isolated from plasma derived from both samples from patients with trauma (*P* < .05) and ovarian cancer (*P* < .005) when compared to HC plasma. However, Figure 7C shows that the concentrations of 5G9-captured TF(IIID8)-AF647-labeled EVs are not different between healthy (n = 18), trauma (n = 8), and ovarian cancer PFP (n = 11). Similarly, Figure 7B shows that the concentrations of TF(IIID8)-AF647–labeled EV captured on the isotype are not different between healthy (n = 18), trauma (n = 8) and ovarian cancer PFP (n = 11). Supplementary Figure S3 shows that the concentrations of TF(IIID8)-AF647–labeled EV captured on CD81 are not different. Supplementary Figure S3 also shows representative images of TF(IIID8)-AF647 fluorescent labeling of EV in healthy trauma and ovarian cancer PFP, stratified by antibody capture spot.

**Figure 8:**
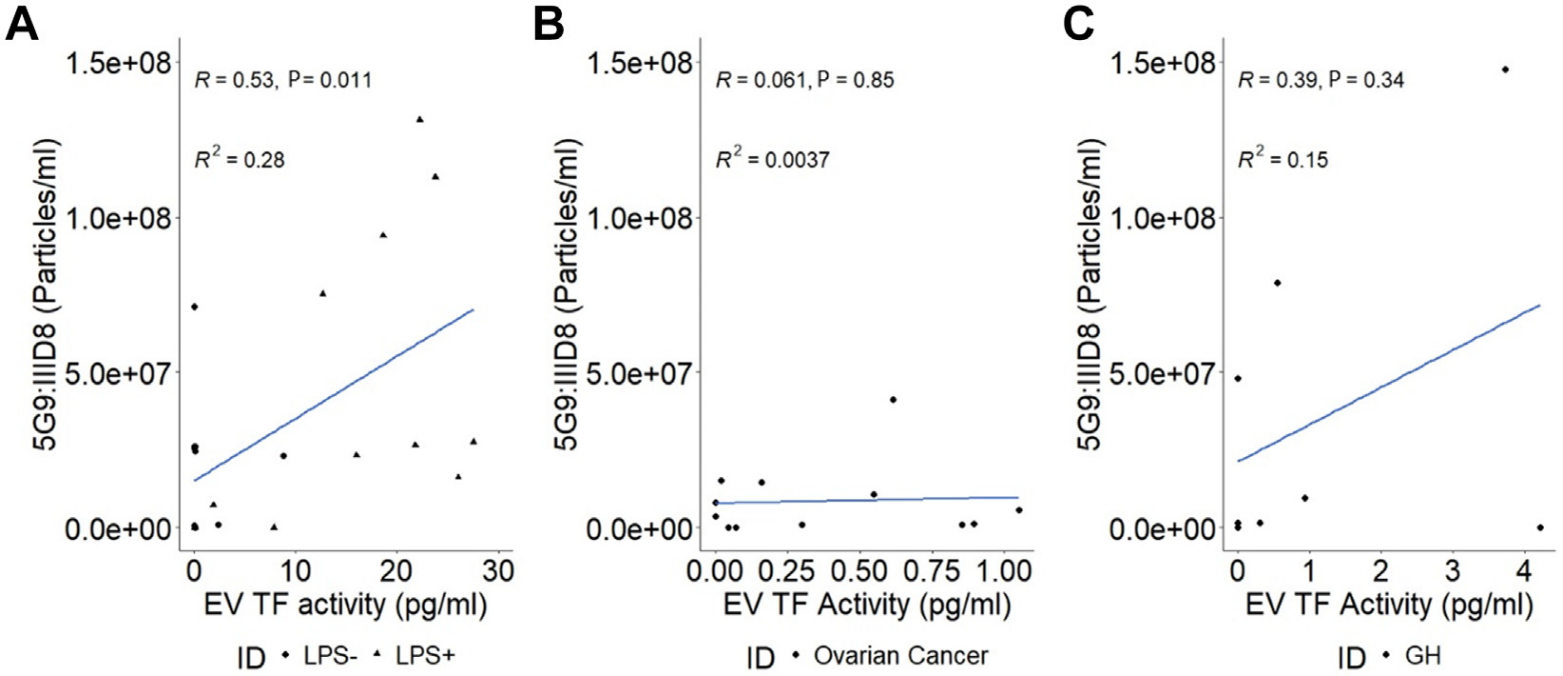
Tissue factor (TF) correlations between ExoView measurements and TF activity assay. (A) 5G9 captured IIID8-labeled extracellular vesicle (EV) count’s relationship with EV TF activity (R^2^ = 0.28, *P* = .011, N = 22) (11 lipopolysaccharide^−^, 11 lipopolysaccharide^+^). (B) Relationship of 5G9-captured IIID8-labeled EV from patients with ovarian cancer with EV TF activity (R^2^ = 0.0081, *P* = .77, N = 12). (C) Relationship of 5G9-captured IIID8-labeled EVs from trauma patients with EV TF activity (R^2^ = 0.15, *P* = .34, N = 8). LPS, lipopolysaccharide.

Figure 8 shows correlations between antigenic fluorescent TF^+^ EV measurements made between different TF antibody capture spots compared with EV TF activity. Figure 8A shows that there is a positive relationship between 5G9-captured TF(IIID8)-AF647-labeled EVs and TF activity in LPS^-^ and LPS^+^ PFP (R^2^ = 0.28, *P* < .05). Figure 8B, C shows no significant association between 5G9-captured TF(IIID8)-AF647 in LPS^−^/LPS^+^ PFP in trauma PFP (Figure 8B) or ovarian cancer PFP (Figure 8C).

## 4 Discussion

The ExoView R100 system has been used in characterizing EVs in health, disease, and cell culture [[36], [37], [38]]. Most literature on ExoView focuses on capture and detection of EVs based on tetraspanin expression, including CD63, CD9, and CD81 [36]. There are some novel uses of ExoView in identifying markers of interest. For instance, following tetraspanin capture, CD14 has been shown to be a marker of acute respiratory distress syndrome [32]. Phosphatidylserine has also been measured following capture on standard chips [31].

Antigenically measuring TF^+^ EV in plasma has been historically challenging. The low concentration of TF^+^ EV in plasma often falls under the detection limit of conventional antigenic assays [[15], [16], [17], [18]]. Furthermore, flow cytometry is often incapable of resolving small EV [28]. TF measurement also lacks a gold standard assay and a well-defined, reproducible standard. Despite these issues, several TF enzyme-linked immunosorbent assays (ELISAs) have been developed and, in some cases, compared against one another, normally with nonplasma samples [44,48]. They often fail to detect TF in plasma [19,20]. Despite these issues, roadmaps have been built to pave the way toward antigenic TF EV detection in plasma [26,27]. We hypothesized that the most promising sandwich ELISA combinations combined with ExoView technology may allow for direct capture and quantification of TF EV [44,48]. We first tested IIID8-AF647, VIC12-AF647, and 5G9-AF647 by monocyte flow cytometry. These data showed a significant increase in TF expression and MFI after 5 hours of LPS stimulation with all 3 antibodies. As with previous studies, there are high and low responders to LPS stimulation [59,60]. In contrast to the existing literature, some of the nonstimulated monocytes appear to be expressing more background TF than typically expected. Normal ranges for unstimulated monocytes are under 1.5%; however, our data show that 21.3% (IIID8), 9.5% (VIC12), and 21.8% (5G9) of monocytes express TF [59]. The specificity of IIID8 and 5G9 has been shown before [48,61,62]. It is possible that time delays in processing blood for monocyte TF measurements may have contributed to the increased expression in TF in some of the samples. It is important to state that the LPS− control PFP samples for ExoView and TF^+^ EV samples were processed immediately without any delays to minimize any potential induction of TF expression by monocytes and release of TF^+^ EV. IIID8-AF647 showed the greatest MFI difference between LPS− and LPS+ samples and was, therefore, chosen for fluorescent detection with ExoView. We showed (by use of CAT) that in the absence of cells, there is TF activity associated with EVs. In line with previous studies [2], we showed that lag time and time to peak are significantly reduced in LPS+ PFP compared with that in LPS− PFP. We also showed that pretreatment of LPS+ plasma with inhibitory anti-TF antibody (HTF-1) partially ablates the reductions in lag time and time to peak. Furthermore, the removal of EV through double centrifugation at 20,000 × *g* fully reverses the reduced lag time and time to peak of LPS+ samples. The partial reduction in thrombin generation observed with HTF-1 pretreatment compared with full reversal following double centrifugation at 20,000 × *g* may have been due to HTF-1 competing with FVII/FVIIa for binding to TF [42]. FVII/FVIIa is present in plasma and has high affinity for TF similar to an antibody binding [42].

Cell culture supernatant derived from serum-starved cells is a compatible biofluid for NTA analysis. However, NTA is not capable of determining if these BxPC-3 EVs were TF^+^. Interestingly, in line with a previous publication [29], the recorded size of EV with ExoView was considerably smaller than that with NTA. ExoView size measurements have been shown to align with transmission electron microscopy size results, whereas NTA is subject to broader size distributions and substantially larger recorded sizes [29].

Only a combination of monoclonal antibodies that bind at distinct epitopes can be used for TF detection in the form of sandwich ELISAs [44,48]. Combining IIID8-AF647 with an anti-TF antibody 5G9 capture system pulled down TF EV and allowed for their measurement in BxPC-3 cell culture supernatant and LPS-stimulated PFP. To our knowledge, this is the first use of ExoFlex technology for customized capture and detection of a rare EV population. Despite promising results with LPS+ stimulated samples, IIID8-AF647 labeling of 5G9-captured TF^+^ EV concentration was not increased either in trauma or ovarian cancer samples. It was also interesting that TF expression increased in LPS+ samples capture by CD81. HTF-1 capture of TF-expressing EVs was also tested and showed efficacy within BxPC-3 cell culture supernatant but not LPS+ PFP. The most likely explanation for this result is that FVII/FVIIa in plasma competes with HTF-1 for binding to TF.

The LPS^−/+^ PFP EV TF activity varied from 0 to 30 pg/mL. Over this range there was a significant association between EV TF activity and the 5G9 captured, IIID8-AF647 labeled EV concentration. EV TF activity in the trauma and ovarian cancer cohorts was significantly higher than that in HCs. In trauma EV TF activity varied from 0 to 4 pg/mL and the association with 5G9 captured, IIID8-AF647–labeled EV concentration was not significant. Furthermore, in ovarian cancer, where EV TF activity varied from 0 to 1 pg/mL, the relationship with 5G9-captured IIID8-AF647-labeled EV concentration was not significant.

These data indicate that although antigenic detection of TF^+^ EV in plasma is possible, the sensitivity was not sufficient to detect TF^+^ EVs in clinical samples. At present, the threshold for utility of antigenic TF^+^ EV detection in plasma by ExoView has not been established, but we estimate that an association exists above 5 pg/mL of TF activity. Interestingly, there is an antigenic signal measured by ExoView that clusters at 0 pg/mL, across all cohorts measured. This could indicate a failure of antigenic detection or even represent a pool of encrypted TF^+^ EVs with no activity. Encrypted TF^+^ EVs may represent a latent pool of potential TF activity [63].

A limitation of this study is the small number of clinical samples available for use across all the assays employed. However, this work does allow for the establishment of ad hoc power tests for future work using ExoView on future cohorts of patients. We are also limited in our ability to provide data on sociocultural determinants across participants, which may be a relevant factor. It is also worth considering that other approaches may be beneficial. A recent publication showed effective immunomagnetic isolation of EVs from plasma via CD29 and CD59 capture and found that levels of EV TF activity were comparable to the TF activity of EVs isolated by centrifugation [33]. Our data show the potential for TF^+^ EV detection using ExoView technology.

## 5 Conclusions

We show proof of principle that TF^+^ EV detection is possible in plasma using ExoView technology, but the exact threshold and clinical utility have yet to be established. There is also further need to establish whether alternate methods could improve the sensitivity and specificity of TF^+^ EV detection using ExoView technology.
