# Supplementary material for: Detection of tissue factor–positive extracellular vesicles using the ExoView R100 system
Source: Res Pract Thromb Haemost. 2023 May 16;7(4):100177. doi: 10.1016/j.rpth.2023.100177 (PMC10276261; doi:10.1016/j.rpth.2023.100177)
Supplement: Supplementary Figure [file mmc1.docx]

# Supplementary Materials

## Supplementary methods

### TF antibody processing and conjugation

Anti-human TF antibodies 5G9 (a gift from Dr. James Morrissey) and IIID8 [41] were obtained in unconjugated forms. The protein concentration of each antibody was confirmed upon receipt using BCA assay (Pierce BCA Protein Assay Kit). Each antibody was purified by immunoprecipitation using a BSA removal kit (AbCam Cambridge, UK). Briefly, BSA removal buffer was incubated at 37°C for 10 minutes. BSA removal buffer was added to antibody at a ratio of 1:1.25. Each BSA removal buffer:antibody mixture was vortexed and incubated for 5 minutes at room temperature. Mixtures were centrifuged at 12,000 x g for 5 minutes twice, to ensure a pellet formed. Supernatant was aspirated and the pellet was resuspended in 500 μl of 1 μm filtered PBS.

Ultra-0.5ml centrifugal filter units (Amicon, Harrogate, UK) were used to concentrate antibodies. 500 μl of sample was added to the filter device placed inside filtrate collection tubes. Centrifugal filter units were centrifuged at 14,000 x g for 30 minutes at room temperature. The filter device was removed, inverted and place inside a new filtrate collection tube. Filter units were then centrifuged twice at 1000 x g for 10 minutes, the resultant liquid (15 μl) was highly concentrated, purified antibody.

Following BCA of purified antibody, IIID8 was diluted to 1mg/ml for conjugation to AF647 following manufacturer recommendations (Lightning-Link^®^ Rapid Alexa Fluor^®^ 647 Labelling Kit, Abcam). Briefly, 1 μl of modifier reagent was added to 10 μl of antibody, gently mixed and added to lyophilised AF647. IIID8-modifier-AF647 were incubated in the dark at room temperature for 30 minutes. After incubation, 1 μl of quencher reagent was added and mixed gently. Conjugated IIID8-AF647 was diluted 1:5 to a total volume of 50 μl. Sodium azide (Sigma Aldrich) was added at a final concentration of 0.01% and stored at 4°C.

To allow for EV capture experiments, ExoFlex kits (Unchained Labs, Pleasanton, CA, USA) were used. 5G9 was diluted to 0.5 mg/ml. 8 μl of ExoFlex modifier was added to 80 μl of antibody and gently mixed. ExoFlex linker modifier-antibody (5G9) mixture was then added to lyophilised ExoFlex Linker 1 and incubated for 45 minutes at room temperature. 8 μl of ExoFlex quencher was then added and incubated for 15 minutes at room temperature. Conjugated antibody was stored at 4°C.

## Supplementary Results

|  | Patients (n= 14) |
| --- | --- |
| Age, years | 37 (19-79) |
| Male, n | 14 |
| Injury severity score | 25 (9-66) |
| New injury severity score | 33 (9-66) |
| Traumatic brain injury, n | 1 |
| Extracranial injury, n | 7 |
| Extracranial injury and traumatic brain injury, n | 6 |
| Race and Ethnicity | Not recorded |

**Table 1- Trauma cohort demographics. Data expressed as mean (range)**

|  | Patients (n= 12) |
| --- | --- |
| Age, years | 55 (26-78) |
| Male, n | 0 |
| Cancer Grade 0 | 1 |
| Cancer Grade 3 | 11 |
| Race and Ethnicity | Not recorded |

**Table 2- Ovarian cancer cohort demographics. Data expressed as mean (range)**


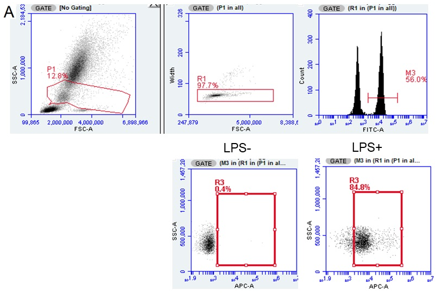


**Figure 1-Flow cytometry gating strategy A) Flow cytometry gating strategy of monocytes (unstimulated and LPS stimulated) from citrated whole blood. Monocytes were selected based upon forward scatter, side scatter and removal of doublets. From this gate, CD14-FITC+ positive monocyte events were selected. Anti-tissue factor (TF) antibody (IIID8-AF647, VIC12-AF647, or 5G9-AF647) was used to identify TF positive events. Prior to labelling, LPS stimulated blood was treated with 10 mg/ml lipopolysaccharide for 5 hours at 37^O^C whilst being gently shaken.**


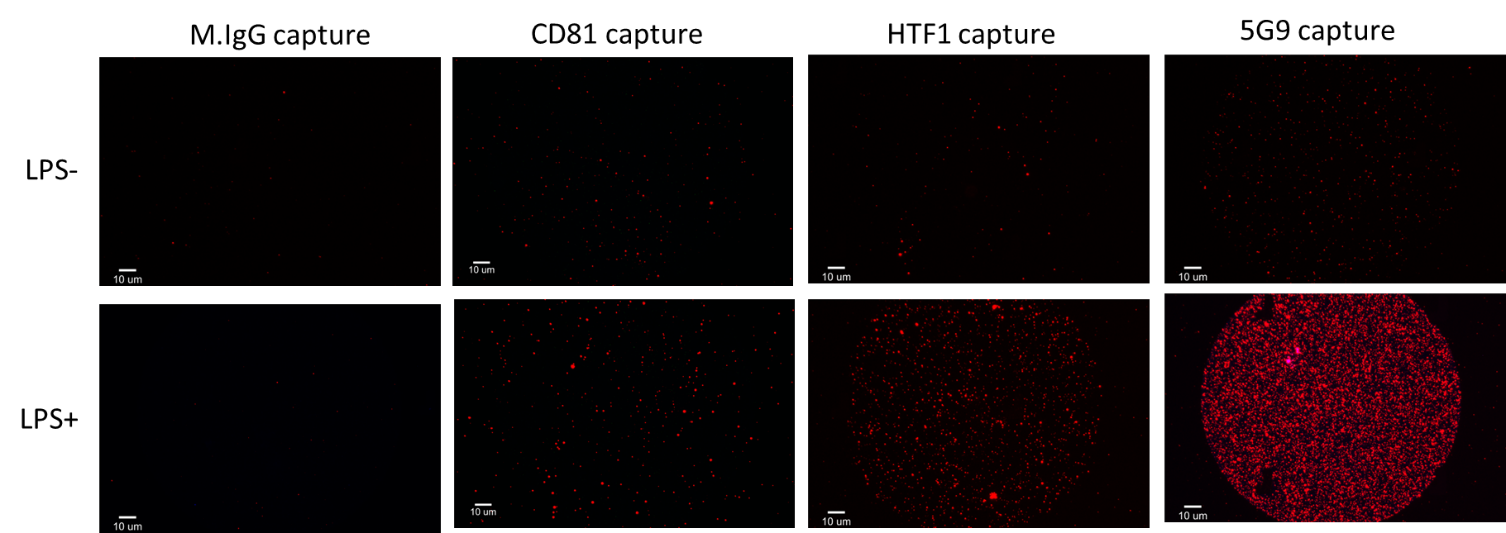


Figure 2- Representative images of EVs captured and labelled with TF(IIID8)-AF647


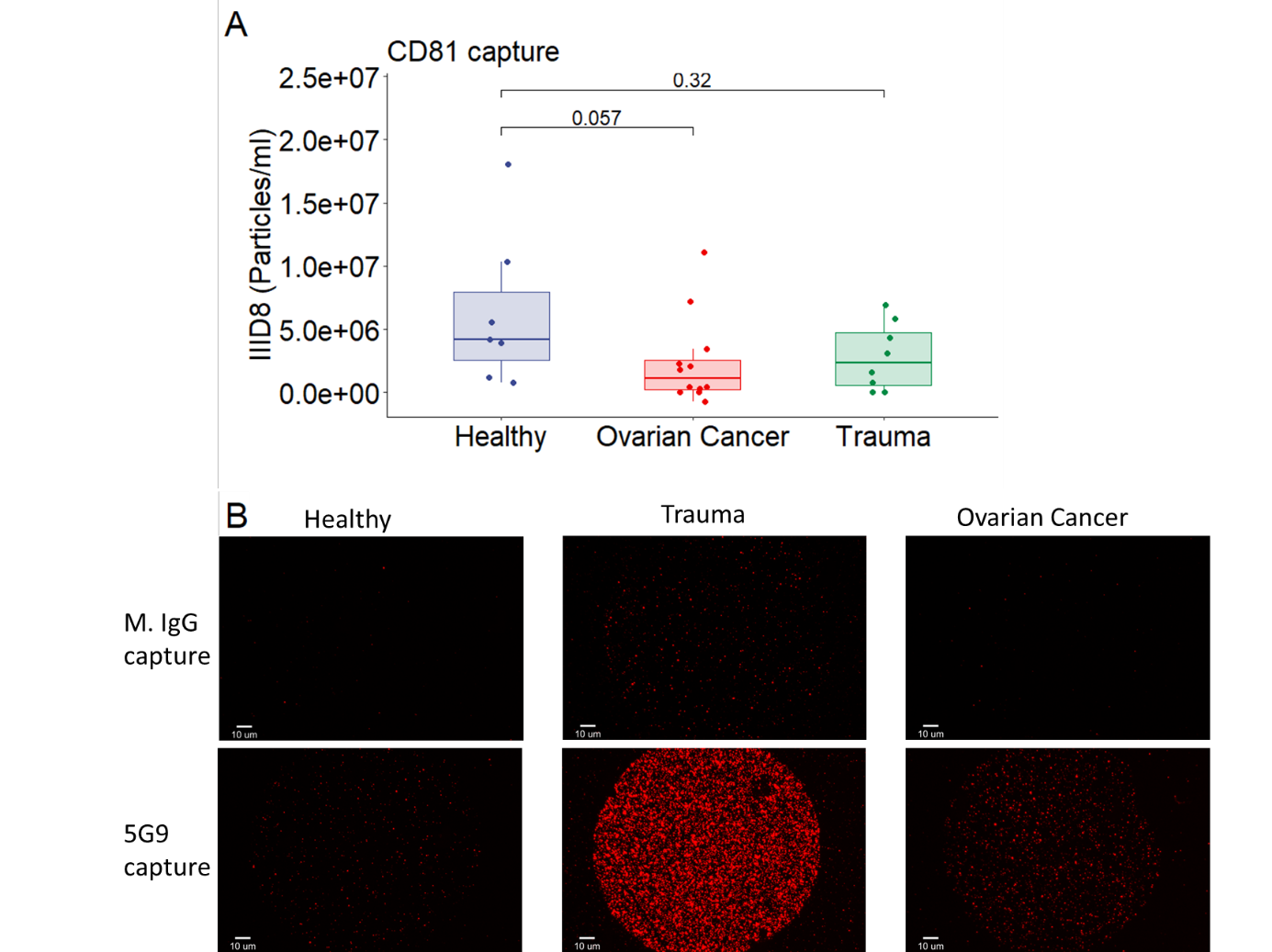


**Figure 3- EV captured and labelled with TF(IIID8)-AF647 A) ExoView fluorescent extracellular vesicle (EV) count anti-tissue factor (TF) antibody (IIID8-AF647) on platelet-free plasma (PFP) with capture based on CD81 antibody. Samples were derived from trauma patients within 1 hour of injury (p = 0.32, N = 8) and ovarian cancer patients (p = 0.057, N = 12) compared against healthy controls. B) Representative images of TF (IIID8-AF647) labelling of EV stratified based on antibody capture (Mouse IgG, TF(5G9)) using plasma samples derived from trauma patients within 1 hour of injury, ovarian cancer and healthy controls.**
